# Promoting the Assessment of Physical Activity and Cardiorespiratory Fitness in Assessing the Role of Vascular Risk on Cognitive Decline in Older Adults

**DOI:** 10.3389/fphys.2019.00670

**Published:** 2019-05-31

**Authors:** David Jiménez-Pavón, Ana Carbonell-Baeza, Carl J. Lavie

**Affiliations:** ^1^MOVE-IT Research Group and Department of Physical Education, Faculty of Education Sciences, University of Cádiz, Cádiz, Spain; ^2^Biomedical Research and Innovation Institute of Cádiz (INiBICA) Research Unit, Puerta del Mar University Hospital, University of Cádiz, Cádiz, Spain; ^3^Department of Cardiovascular Diseases, John Ochsner Heart and Vascular Institute, Ochsner Clinical School - The University of Queensland's School of Medicine, New Orleans, LA, United States

**Keywords:** dementia - Alzheimer disease, cognitive decline and dementia, physcial activity, cardiorespiratory fitness (CRF), aging, older adult

## Introduction

The world population has been experiencing significant aging processes that have resulted in rising proportions of older persons in the total population since the mid-twentieth century. It is estimated that by 2025 there will be 1.2 billion people over 60 years of age and by 2050 there will be 2 billion, with 80% in developing countries (World Health Organization, 2011) Moreover, the recent (Alzheimer's Disease International., 2018) indicates that over 50 million people live with dementia worldwide, which is expected to exceed 152 million by 2050. There is no known cure for dementia and, thus, preventative strategies are of great importance. In fact, the annual cost of dementia care was expected to exceed $1 trillion by 2018, thereby, primary prevention is currently considered a potential tool to delay the disease onset by modulating modifiable risk factors. In fact, it has been suggested around 35% of dementia is attributable to a combination of nine modifiable risk factors, including education, hypertension, obesity, hearing loss, depression, diabetes, physical inactivity, smoking, and social isolation (Livingston et al., 2017). In line with that, three potential brain mechanisms through which preventative strategies may reduce dementia risk are: (a) reduced brain damage (vascular, neurotoxic and oxidative stress), (b) reduced brain inflammation, and (c) increased brain cognitive reserve with greater physical activity (PA) and exercise suggested as a common factor to promote all these mechanisms (Livingston et al., 2017). Therefore, greater PA and fitness (especially cardiorespiratory fitness, CRF) are currently considered key factors to promote in the prevention of cognitive decline and dementia (Kennedy et al., 2017; Livingston et al., 2017; Müller et al., 2017). Moreover, these key factors not only directly affecting cognitive decline and dementia, but also have effect through their mediation effects on other related risk factors, such as hypertension, obesity, depression, and diabetes (Chase et al., 2009; Kokkinos et al., 2017; Celis-Morales et al., 2018; Chekroud et al., 2018; Ortega et al., 2018).

Particularly well-established in the literature is the role of PA and CRF on cardiovascular (CV) risk factors and diseases (CVD), as well as mortality (Blair et al., 1998; Jiménez-Pavón et al., 2016; Ortega et al., 2018). However, studies analyzing the role of vascular risk on cognitive decline have been based solely upon the use of the Framingham Heart Study general cardiovascular disease score (i.e., Framingham Risk Score (FRS) (Eldholm et al., 2018; Rabin et al., 2018), and no studies have investigated the potential mediation effect of PA and CRF on the vascular risk of cognitive decline and dementia. Hence, we believe that to better understand the relationship between vascular risk and cognitive decline, assessment of PA and CRF should be included when determining an individual's vascular risk factors. Therefore, the purpose of this opinion article is to encourage the assessment of PA and fitness, at least CRF, when studying the role of vascular risk on cognitive decline.

## Implications of PA and CRF in the Approach to Cognitive Decline

Rabin et al. (2018), in their study on the interactive associations of vascular risk and β-amyloid (Aβ) burden with cognitive decline, concluded that FRS and other vascular risks (imaging biomarkers including β-amyloid, hippocampal volume, fludeoxyglucose F18–labeled positron emission tomography, and white matter hyperintensities) were associated with longitudinal cognitive decline, suggesting that vascular risk may complement other imaging biomarkers in assessing the risk of cognitive decline in older adults with preclinical Alzheimer disease. We believe their study is highly relevant, but important considerations on the role that modifiable risk factors, such as PA and CRF, have on vascular risk, cognitive decline, and its relationships, are needed.

The mentioned study uses the FRS as a good indicator of vascular risk and also, in fact, for coronary heart disease (CHD) and mortality (Gander et al., 2015). They found that the FRS was associated with cognitive decline, both alone and synergistically, with Aβ burden. However, other lifestyle factors, such as PA levels, especially those which improve CRF, and CRF itself, have proven to be important determinants of vascular risk in terms of CHD and mortality (Gander et al., 2015). On the other hand, PA and CRF have also been stated in the last years to be important markers related with cognitive status/decline, Alzheimer disease (Livingston et al., 2017; Schultz et al., 2017; Rosenberg et al., 2018), and other parameters directly related with brain health (i.e., body mass index, blood pressure, fasting glucose or BDNF) (Rosenberg et al., 2018). Moreover, the hypothesis that regular exercise, and specifically regular exercise associated with improved CRF, enhances cognition through the beneficial effects of exercise on vascular health has been recently highlighted (Barnes and Corkery, 2018).

On the one hand, Halloway et al. (2018) found that higher levels of total daily PA, assessed by accelerometer, was significantly related to larger gray matter volumes, including subcortical gray matter and total gray matter. Moreover, Arenaza-Urquijo et al. (2017) showed regional associations of PA with gray matter in the prefrontal, insular and motor cortices and the anterior part of the hippocampus after adjustment for confounders (age, sex, years of education and body mass index). Thus, engagement in PA in late adulthood was independently related to regional gray matter volume, notably in aging and Alzheimer disease vulnerable areas.

On the other hand, high levels of CRF are important for brain health and aging (Barnes and Corkery, 2018). In this line, higher CRF in women at midlife was associated with lower risk of dementia 44 years later (Horder et al., 2018). Moreover, greater CRF levels at age 18 were associated with a lower hazard ratio for the development of mild cognitive impairment or dementia (Nyberg et al., 2014). When we focus on long-term changes in CRF as a consequence exercise intervention and its effectiveness on improving cognition the relevance of CRF is even higher (Barnes and Corkery, 2018). Thereby, a study has shown that the magnitude of change in CRF after intervention, rather than the dose of exercise, was a better predictor of cognitive improvement (Vidoni et al., 2015). This highlights the importance of exercise interventions and PA behaviors achieving improvements in CRF to also benefits cognitive function. Consequently, physical activity and exercise appear to mitigate brain atrophy rates (ie: gray matter, hippocampal, whole brain volumes) which are considered indicators of cognitive decline, however, another factor that could explain some of the beneficial effects of PA and regular exercise on cognition is the maintenance of cerebral blood flow (CBF) (Barnes and Corkery, 2018). Hence, PA and CRF are currently considered key factors of prevention strategies in the early stages of dementia or cognitive decline based on longitudinal and intervention finding (Barnes and Corkery, 2018; Rosenberg et al., 2018).

Therefore, we believe the evidence supports (Barnes, 2015; Barnes and Corkery, 2018) that PA levels and CRF are important determinants in the interactions among vascular risk, Aβ burden and cognitive decline. In this sense, the work of Rabin et al. (2018) would have been a good opportunity to analyze mediation roles of PA and CRF on the evolution of cognitive status through aging USING such a large-scale study, contributing additional evidence of the benefits of prevention strategies focused on modifiable determinants of dementia, such as PA and CRF. On the other hand, Ngandu et al. (2015) analyze the effect of a multidomain intervention (diet, exercise, cognitive training, and vascular risk monitoring) to prevent cognitive decline. However, the particular role of PA and CRF at baseline was not studied. Thus, future studies considering the specific mediation role of PA and CRF are recommended.

## Instruments for Assessing PA and CRF

Ideally, instruments allowing the objective assessment of PA and CRF are desired. The most used method for objective measurement of PA is based on accelerometer technology (Buchman et al., 2012), while adapted incremental exercise tests are appropriate for CRF assessment (Müller et al., 2017) including both definitions, VO_2peak_ and VO_2max_ (Green and Askew, 2018). However, when objective assessments are not possible, at least to include self-reported or indirect measures of both PA (ie., Global Physical Activity Questionnaire-GPAQ) and CRF (i.e., eCRF, estimation based on 7 items) are required and need to be promoted (Armstrong and Bull, 2006; Artero et al., 2014). In fact, it would be of great interest if studies, like the one performed by Rabin et al. (2018), included PA and/or CRF in their collected data to reanalyse the results and consider the role of them, or to consider including these factors as new measurements in future appointments of the ongoing project.

We realize that the proper and habitual way to measure PA and CRF with accuracy implies the use of sophisticated and time-consuming methods, like accelerometers and incremental exercise test protocols, which could hamper its implementation in epidemiological and longitudinal studies. However, self-reported PA has been widely used at epidemiological level when a more objective measurement has not been available (Lee, 2018). In addition, recently it has been proven that estimated-CRF (eCRF), based on information easy to obtain (i.e., eCRF, estimated on the basis of sex, age, body mass index, waist circumference, resting heart rate, physical activity level and smoking status) adds to the clinical assessment of 10-year CHD risk in asymptomatic men (Artero et al., 2014; Gander et al., 2017). In these studies, authors found that among men with “low” 10-year FRS predicted CHD risk, those with high eCRF had a lower risk for CHD compared to men with low eCRF. These results suggest that assessing eCRF from research data or clinical information that is easy to obtain may considerably add to the clinicians' overall risk assessment using FRS (Gander et al., 2017). Thus, eCRF could be a good alternative to objective CRF measurement as it can be quickly and easily collected in epidemiological studies and implemented in the assessment of lifestyle factors with Aβ burden, cognitive decline with aging, and Alzheimer disease. The limitations associated with the use of self-reported PA and indirect assessment of CRF should be made clear because, in general, their precision cannot be equal to that of objective measures, however, when direct measures are not possible the use of indirect measures is highly advisable.

## Conclusion

We encourage authors focused on this interesting research topic for future studies to analyse and/or to include the measurement of PA and CRF, using the most accurate methodology able to them, in order to provide very useful information on their mediation role on vascular risk and its associations with cognitive decline in elderly.

## Author Contributions

D-JP, A-CB, and CL conceived the article and wrote the manuscript and contributed to the critical, appraisal of the manuscript.

### Conflict of Interest Statement

The authors declare that the research was conducted in the absence of any commercial or financial relationships that could be construed as a potential conflict of interest.
